# Evaluation of Helicobacter Pylori eradication in pediatric patients by triple therapy plus lactoferrin and probiotics compared to triple therapy alone

**DOI:** 10.1186/1824-7288-38-63

**Published:** 2012-10-31

**Authors:** Salvatore Tolone, Valeria Pellino, Giovanna Vitaliti, Angela lanzafame, Carlo Tolone

**Affiliations:** 1 Division of General and Bariatric Surgery, Second University of Naples, Via Pansini 5, Naples, 80131, Italy; 2 Department of Pediatrics, Second University of Naples, Via L De Crecchio, Naples, 80138, Italy; 3 Department of Medical and Pediatrics Science, Via SSofia n.78, Catania, 95123, Italy

**Keywords:** H.P, H.P. eradication, Children gastritis, Probiotic

## Abstract

**Background:**

To evaluate whether the addition of a probiotic could improve Helicobacter pylori (H.P.) eradication rates and reduce the side effects of treatment in children.

**Methods:**

Between July 2008 and July 2011 all patients with a clinical, laboratory and endoscopic diagnosis of H.P. positive gastritis referred to our Unit were included in the study. Patients suffering from allergy to any of drugs used in the study, with previous attempts to eradicate H.P. and those who received antibiotics, PPIs or probiotics within 4 weeks were excluded from the present study. Patients were randomized into two therapy regimens (group A and B): both groups received standard triple treatment (omeprazole, amoxicillin and clarithromycin) while only group B patients were also given a probiotic (Probinul - Cadigroup). Patients compliance was evaluated at the end of the treatment. Successful eradication was defined as a negative 13 C-urea breath test (C13-ubt) result four weeks after therapy discontinuation.

**Results:**

A total of 68 histopathologically proven H.P.-infection children (32 male and 36 females) were included in the study. All of the patients in both groups used more than 90% of the therapies and no patients were lost at follow up. All side effects were selflimiting and disappeared once the therapy was terminated. Epigastric pain was observed in 6 (17.6%) group A vs 2 (5.8%) group B patients (P<0.05), nausea in 3 (8.8%) group A vs 1 (2.9%) group B patients (P<0.05); vomiting and diarrhea were observed in 2(5.8%) and 8 (23.5%) group A patients, respectively and never in group B (P<0.05). There was no significant difference between the two groups in terms of constipation (5.8% in group A and B). Four weeks after the completion of therapy, 56/68 patients (82.3%) tested negative for H.P. on C13-ubt. H.P. was eradicated in 26 patients (76.4%) in group A and in 30 patients (88.2%) in group B. There was no significantly difference in the rate of H.P. eradication between group A and group B (p=0.1), although the success rate for H.P. eradication was higher in group B than in group A.

**Conclusion:**

The addition of a probiotic formula to triple therapy significantly decreased the frequency of epigastric pain, nausea, vomiting and diarrhea.

## Background

Helicobacter pylori (H.P.) infection is a major cause of chronic gastritis and peptic ulcers and it is a risk factor for gastric malignancies, adenocarcinoma and low grade gastric mucosa associated lymphoid tissue (MALT) lymphoma [1]. According to the Maastricht III consensus report, H.P. eradication is recommended for patients with gastroduodenal ulcer disease, atrophic gastritis, MALT lymphoma, first degree relatives of patients with gastric cancer, patients with unexplained iron deficiency anemia and chronic idiopathic thrombocytopenic purpura.

In both developed and developing countries, H.P. infection is most frequently acquired during childhood, and it is associated with family size, familial clustering, low socioeconomic status and education. The first line treatment for H.P. infection, as recommended by the Maastricht 2–2000 Consensus Report , is a 7–14 days triple therapy which includes amoxicilline, clarithromycin, OR metronidazole and a proton-pump inhibitor (PPI) [2], though new strategies may be required for treatment both in adults and children. As for adults, “infanti” treatment will fail in approximately 10-35% of patients, and H.P. infection will remain resulting from several factors, including nonadherence to therapy related to adverse effects or complicated dosing regimens and increasing antibiotic resistance [3-5]. To overcome this problem, both in adults than in children, alternative and adjuvant therapies have been added to conventional treatment, such as probiotics (PB) [6,7]. Although there is some controversy as to whether supplementation with probiotic improves the H.P. eradication rates [8-10], several meta-analysis and reviews have suggested that probiotics can improve the H.P. eradication rate by approximately 5-10% [10-13]. However, it is evident that not all probiotics are created equal, that the beneficial effects are strain specific, and each strain must be evaluated individually. Besides treatment studies on children are limited by the small number of infected children in each individual center [14-16], therefore this study aimed to evaluate whether the addition of a commercially multi-strain probiotics to a 7 days triple therapy in children could improve H.P. eradication rates and reduce the side effects of treatment.

## Methods

Between July 2008 and July 2011 children referred to the Department of Pediatrics of the University of Naples with dyspeptic complaints such as heartburn, dyspepsia, nausea and epigastric pain, were enrolled in this study. The study was approved by Ethical Committee of the University of Naples.

Pediatric patients with a clinical, laboratory and endoscopic diagnosis of H.P. positive gastritis and the other conditions necessary H.P. eradication for Maastricht III consensus report were included in the study. Exclusion criteria were: 1) allergy to any of drugs used in the study 2) previous attempts to eradicate H.P. 3) receipt of antibiotics, PPIs or probiotics within 4 weeks of the study. Informed consent was obtained from all patients and all positive 13 C-urea breath test (C13-ubt) patients underwent upper endoscopy.

Two samples were taken from the gastric antrum and compass for histologic assessment, and the biopsy specimens were fixed in 10% formalin solution. Preparation were stained with hematoxylin-eosin and modified Giemsa stains and were evaluated according to updated Sydney classification.

H.P. positive patients were randomized into two therapy regimens: patients in group A were given omeprazole (1 mg/kg before breakfast), amoxicillin (50 mg/kg b.i.d. after meals), clarithromycin (15 mg/kg b.i.d. after meals) for 7 days, whereas patients in group B were given the same drugs and a probiotic once a day for 7 days.

Patients were instructed to take PPI 30 mins before breakfast, the antibiotics 5 mins after breakfast and dinner, and PB supplement in the afternoon. The PB supplement was taken in a commercially available form containing 5 × 109 Lactobacillus plantarum, 2 × 109 L. reuterii, 2 × 109 L. casei subsp. rhamnosus, 2 × 109 Bifidobacterium infantis and B. longum, 1 × 109 L. salivarius, 1 × 109 L. acidophilus, 5 × 109 Streptococcus termophilus, and 1 × 109 L. sporogenes (Lactobacillaceae). This PB formula (5g/dayose q.d.) (Probinul - Cadigroup) was selected because it contains high concentrations of a wide range of bacteria, as well as inuline as a prebiotic.

Parents were asked to report any side effects of therapy during the treatment period and were given a possible side effect list, such as epigastric pain, nausea, diarrhea and constipation. Patients compliance was evaluated at the end of the treatment on the basis of diary that patients were asked to fill with pill count and was considered as completed if >90% of the medication had been taken.

Successful eradication was defined as a negative C13-ubt result four weeks after discontinuation of the therapy.

### Statistical analysis

Data were collected prospectically in an electronic database (Excell Microsoft). Fisher’s exact test was carried out to determine the efficacy of the two treatments. Results were considered statistically significant for P values less than 0.05.

## Results

A total of 68 histopathologically proven H.P.-infection children (32 male and 36 females) were included in the study. The mean age of all children was 8,3 +/− 3,4 years.

The patients were randomized into group A (triple therapy n 34 patients) and group B (triple therapy plus probiotic n 34 patients) for H.P. eradication. The age distribution of patients and gender in group A and group B were similar.

All of parents returned the daily diary filled up with side-effects and pill count.

All of the patients in both groups used more than 90% of the therapies and no patients were lost at follow up. The prevalence of epigastric pain, nausea, vomiting and diarrhea was significantly higher (P<0.05) in group A than in group B . There was no significant difference between the two groups in terms of constipation. (Detailed data on side effects are summarized in Table 1).

**Table 1:** Incidence of side effects of the treatment

**Side Effects**	**Group A** (**N**=**34**)	**Group B** (**N**=**34**)	**P value**
*Epigastric pain*	6 (17.6%)	2 (5.8%)	<0.05
*Nausea*	3 (8.8%)	1 (2.9%)	<0.05
*Vomiting*	2 (5.8%)	0	<0.05
*Diarrhea*	8 (23.5%)	0	<0.05
*Constipation*	2 (5.8%)	2 (5.8%)	N.S.

All side effects were self-limiting and disappeared once the therapy was terminated.

**Figure 1:**
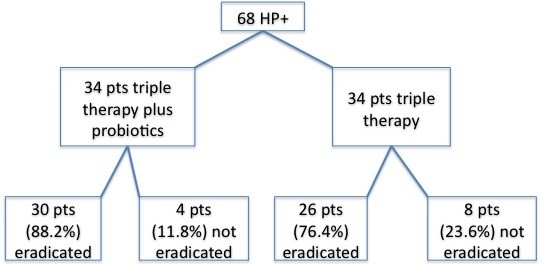
Patients enrollment and therapy flowchart.

Four weeks after the completion of therapy, 56/68 patients (82.3%) tested negative for H.P. on C13-ubt. H.P. was eradicated in 26 patients (76.4%) in group A and in 30 patients (88.2%) in group B (Figure 1). There was no significantly difference in the rate of H.P. eradication between group A and group B (p=0,1), although the success rate for H.P. eradication was higher in group B than in group A.

## Discussion

It is well known that childhood is an important period for acquisition of H.P. infection. Intrafamiliar trasmission of the infection , especially from mother to child, has been hypothisized as the major mode of dissemination [17]. H.P. is considered to be the major cause of chronic gastritis and duodenal ulcer in childhood and an important cofactor in the development of gastric cancer [18]. Unfortunately eradication therapy is not always successful and reports of failed H.P. eradication therapy are increasing. Therefore, recent review studies report eradication rates of standard triple therapy in children below 75% [14,19]. Moreover this regimens have the disadvantage of risking poor compliance and causing side-effects especially in children. Nowadays there is considerable interest in alternative therapies or adjunctive treatment against H.P. to reduce some of the drawbacks associated with the antibiotic consumption and to increase the eradication rates. Some adjuvant therapy trials both in adults and in children incorporate probiotics [20-22].

In our study H.P. eradication was achieved in 82.3% of patients. A little increase in the eradication rate and a significant reduction in side effects were observed in the group treated with triple therapy plus Probinul PB. Resistant patients were offered to second line therapy (omeprazole 1 mg/kg/d, amoxicillin 50 mg/kg/d and metronidazole 20 mg/kg/d), according to NASPGHAN guidelines, confirming the contribution of a high background resistance to clarithromycin. Also, guidelines on HP infection in children issued so far suggest that the antibiotic susceptibility test should be performed whenever available [15]. However, we did not performed susceptibility test for first line antibiotic therapy.

Probiotics include viable microorganism that have a beneficial effects for the prevention and treatment of specific pathological conditions [23]. Principle mechanisms include interference with pathogenic toxins, preservation of cellular physiology , interference with pathogen attachment and interaction with normal microbiota [24,25] .

In addition, stimulation or modulation of immune responses, both within the lumen and systemically, although not clearly linked to H.P. infection, may contribute [26,27]. Besides probiotics may be beneficial in reducing adverse effects and increasing tolerability of HP eradication regimens. Several studies evaluated whether probiotic supplementation might help to prevent or reduce drug-related side effects during H.P. eradication therapy in adults [28-30]. Up to date, in the pediatric population, few randomized studies have evaluated whether consumption of probiotics could increase H.P. eradication rates and reduce the side effects of treatment [31-33]. In fact, in a recent trial by Szajewska et al. [33] was identified that the use of Lactobacillus GG along with standard triple therapy didn’t resulted in an increased eradication rate and decreased overall therapy-related side effects.

Some of these trials do not provide evidence on the beneficial effect in children of supplementation of probiotics to triple therapy for eradicating H.P. infection nor for positively affecting therapy related symptoms and overall treatment tolerance [34].

However other investigators have shown that in symptomatic H.P. positive children, the occurrence of antibiotic associated side-effects was significantly reduced by the addition of probiotics compared with the placebo supplemented group [31].

H.P. eradication depends on a number of factor, including patients compliance, adverse effects, bacterial resistance, poor drug distribution and concentration, socio-economic conditions and geographic differences. So antibiotic related side effects may be depending on different probiotic strains taken during the H.P. eradication therapy.

In our study the choice of the Probinul probiotic formula was determinated by the fact that it contains high concentration of a wide range of bacteria, as well as inuline as prebiotic. Previously we have demonstrated that this probiotic formula was able to reduce antibiotic gastro-intestinal related side effects in children treated with amoxicillin therapy for pneumonia infections [35].

Besides De Bortoli et al. have demonstrated that the addition of bovine lactoferrin and this probiotic formula to standard triple eradication therapy could improve the H.P. eradication rate and reduce side effects in adult [36].

In this study the combination of standard triple therapy + probiotic + bovine lactoferrin was more effective than triple therapy alone. This could be explained by the combined effect of the bactericidal and bacteriostatic properties of bovine lactoferrin and the mechanism of the probiotics including their direct, nonspecific, bacteriostatic activity and their enhancement of immunoglobulin A production [37].

Probiotics may act through both immunological as well as non-immunological mechanism in H.P. eradication. The latest mechanism includes providing antimicrobial substances, competing with H.P. for adhesion and providing a mucosal barrier. When there are decreases in side effects, compliance improves [26]. The efficacy of probiotic supplementation for reducing side effects during the course of anti HP regimens appears to be dependent of which probiotic species are used. In fact probiotics must be metabolically active in the intestinal lumen, where they should survive but not persist after the therapy regimen has been completed. They must be acid and bile resistant and should be antagonist to pathogenic bacteria [38-40].

The present study has shown that the addiction of the Probinul probiotic formula to standard antibiotic treatment reduced in children H.P. therapy-associated side effects (occurrence of side effects: 61.5% in group A vs 14.5% in group B). The H.P. eradication was achieved in 26 of 34 patients in group A (76.4%) and in 30 of 34 patients (88.2%) in group B. Although the success rate was higher in group A than in group B, the different was not significant. Also, we preferred to use the conventional 7-days triple therapy to better highlight the efficacy of probiotic supplement; furthermore extending the duration of therapy form 7 to 14 days is not clearly associated with an increased eradication rate [41]. However, our study presents some limitations, as a relative small number of patients and absence of a placebo-control group for the probiotics; also, the treatment group with probiotics is likely to be superior to the control group if the sample size is larger.

In conclusion our study suggests that the addition of this probiotic formula to triple therapy did not increase (significantly) the H.P. eradication rates; however it significantly decreased the frequency of epigastric pain, nausea, vomiting and diarrhea.

In children with H.P. infection, we think, there is evidence to recommend the use of this probiotic formula along with standard triple therapy as an option for decreasing overall therapy related side effects and slightly increasing the eradication rates.

## Competing interests

The authors declare that they have no competing interests.

## Authors’ contributions

TS designed the study and wrote the manuscript; PV, VG and LA provided data collection and were involved in editing the manuscript; TC designed the study, wrote the manuscript and gave final approval of the version to be published. All authors read and approved the final manuscript.

## References

[B1] Suerbaum S Michetti P Helicobacter pylori infection N Engl J Med 2002 347 1175 1186 10.1056/NEJMra020542 12374879

[B2] Malfertheiner P Mégraud F O'Morain C Hungin AP Jones R Axon A Graham DY Tytgat G European Helicobacter Pylori Study Group (EHPSG) Current concepts in the management of Helicobacter pylori infection–the Maastricht 2–2000 Consensus Report Aliment Pharmacol Ther 2002 16 167 180 11860399 10.1046/j.1365-2036.2002.01169.x

[B3] Chey WD Wong BC Practice Parameters Committee of the American College of Gastroenterology American College of Gastroenterology guideline on the management of Helicobacter pylori infection Am J Gastroenterol 2007 102 1808 1825 10.1111/j.1572-0241.2007.01393.x 17608775

[B4] Malfertheiner P Megraud F O'Morain C Bazzoli F El-Omar E Graham D Hunt R Rokkas T Vakil N Kuipers EJ Current concepts in the management of Helicobacter pylori infection: the Maastricht III Consensus Report Gut 2007 56 772 781 10.1136/gut.2006.101634 17170018 PMC1954853

[B5] Mamori S Higashida A Kawara F Ohnishi K Takeda A Senda E Ashida C Yamada H Age-dependent eradication of Helicobacter pylori in Japanese patients World J Gastroenterol 2010 16 4176 4179 10.3748/wjg.v16.i33.4176 20806435 PMC2932922

[B6] Gotteland M Brunser O Cruchet S Systematic review: are probiotics useful in controlling gastric colonization by Helicobacter pylori? Aliment Pharmacol Ther 2006 23 1077 1086 10.1111/j.1365-2036.2006.02868.x 16611267

[B7] Tong JL Ran ZH Shen J Zhang CX Xiao SD Meta-analysis: the effect of supplementation with probiotics on eradication rates and adverse events during Helicobacter pylori eradication therapy Aliment Pharmacol Ther 2007 25 155 168 17229240 10.1111/j.1365-2036.2006.03179.x

[B8] Ozdil K Calhan T Sahin A Senates E Kahraman R Yǘzbasioglu B Demirdag H Demirsoy H Sökmen MH Levofloxacin based sequential and triple therapy compared with standard plus probiotic combination for Helicobacter pylori eradication Hepatogastroenterology 2011 58 1148 1152 10.5754/hge11075 21937367

[B9] Tursi A Brandimarte G Giorgetti GM Modeo ME Effect of Lactobacillus casei supplementation on the effectiveness and tolerability of a new second-line 10-day quadruple therapy after failure of a first attempt to cure Helicobacter pylori infection Med Sci Monit 2004 10 CR662 CR666 15567983

[B10] Medeiros JA Gonçalves TM Boyanova L Pereira MI de Carvalho JN Pereira AM Cabrita AM Evaluation of Helicobacter pylori eradication by triple therapy plus Lactobacillus acidophilus compared to triple therapy alone Eur J Clin Microbiol Infect Dis 2011 30 555 559 10.1007/s10096-010-1119-4 21207091

[B11] Szajewska H Horvath A Piwowarczyk A Meta-analysis: the effects of Saccharomyces boulardii supplementation on Helicobacter pylori eradication rates and side effects during treatment Aliment Pharmacol Ther 2010 32 1069 1079 10.1111/j.1365-2036.2010.04457.x 21039671

[B12] Chenoll E Casinos B Bataller E Astals P Echevarría J Iglesias JR Balbarie P Ramón D Genovés S Novel probiotic Bifidobacterium bifidum CECT 7366 strain active against the pathogenic bacterium Helicobacter pylori Appl Environ Microbiol 2011 77 1335 1343 10.1128/AEM.01820-10 21169430 PMC3067243

[B13] Wilhelm SM Johnson JL Kale-Pradhan PB Treating bugs with bugs: the role of probiotics as adjunctive therapy for Helicobacter pylori Ann Pharmacother 2011 45 960 966 10.1345/aph.1Q104 21693698

[B14] Francavilla R Lionetti E Castellaneta SP Magistà AM Boscarelli G Piscitelli D Amoruso A Di Leo A Miniello VL Francavilla A Improved efficacy of 10-Day sequential treatment for Helicobacter pylori eradication in children: a randomized trial Gastroenterology 2005 129 1414 1419 10.1053/j.gastro.2005.09.007 16285942

[B15] Oderda G Shcherbakov P Bontems P Urruzuno P Romano C Gottrand F Gómez MJ Ravelli A Gandullia P Roma E European Pediatric Task Force on Helicobacter pylori Results from the pediatric European register for treatment of Helicobacter pylori (PERTH) Helicobacter 2007 12 150 156 10.1111/j.1523-5378.2007.00485.x 17309752

[B16] Lionetti E Indrio F Pavone L Borrelli G Cavallo L Francavilla R Role of probiotics in pediatric patients with Helicobacter pylori infection: a comprehensive review of the literature Helicobacter 2010 15 79 87 10.1111/j.1523-5378.2009.00743.x 20402810

[B17] Kivi M Tindberg Y Sörberg M Casswall TH Befrits R Hellström PM Bengtsson C Engstrand L Granström M Concordance of Helicobacter pylori strains within families J Clin Microbiol 2003 41 5604 5608 10.1128/JCM.41.12.5604-5608.2003 14662948 PMC309035

[B18] Uemura N Okamoto S Yamamoto S Matsumura N Yamaguchi S Yamakido M Taniyama K Sasaki N Schlemper RJ Helicobacter pylori infection and the development of gastric cancer N Engl J Med 2001 345 784 789 10.1056/NEJMoa001999 11556297

[B19] Kato S Konno M Maisawa S Tajiri H Yoshimura N Shimizu T Toyoda S Nakayama Y Iinuma K Results of triple eradication therapy in Japanese children: a retrospective multicenter study J Gastroenterol 2004 39 838 843 10.1007/s00535-004-1398-6 15565402

[B20] Song MJ Park DI Park JH Kim HJ Cho YK Sohn CI Jeon WK Kim BI The effect of probiotics and mucoprotective agents on PPI-based triple therapy for eradication of Helicobacter pylori Helicobacter 2010 15 206 213 10.1111/j.1523-5378.2010.00751.x 20557362

[B21] Kim MN Kim N Lee SH Park YS Hwang JH Kim JW Jeong SH Lee DH Kim JS Jung HC Song IS The effects of probiotics on PPI-triple therapy for Helicobacter pylori eradication Helicobacter 2008 13 261 268 10.1111/j.1523-5378.2008.00601.x 18665934

[B22] Chiesa C Pacifico L Anania C Poggiogalle E Chiarelli F Osborn JF Helicobacter pylori therapy in children: overview and challenges Int J Immunopathol Pharmacol 2010 23 405 416 20646336 10.1177/039463201002300203

[B23] Jonkers D Stockbrűgger R Review article: Probiotics in gastrointestinal and liver diseases Aliment Pharmacol Ther 2007 26 133 148 18081657 10.1111/j.1365-2036.2007.03480.x

[B24] Leonardi S Miraglia del Giudice M La Rosa M Bellanti J Atopic disease, immune and environment Allergy Asthma Proc 2007 28 410 417 10.2500/aap.2007.28.2954 17883908

[B25] del Giudice MM Rocco A Capristo C Probiotics in the atopic march: highlights and new insights Dig Liv Dis 2006 38 S288 S290 10.1016/S1590-8658(07)60012-7 17259093

[B26] McFarland LV Systematic review and meta-analysis of Saccharomyces boulardii in adult patients World J Gastroenterol 2010 16 2202 2222 10.3748/wjg.v16.i18.2202 20458757 PMC2868213

[B27] Franceschi F Cazzato A Nista EC Scarpellini E Roccarina D Gigante G Gasbarrini G Gasbarrini A Role of probiotics in patients with Helicobacter pylori infection Helicobacter 2007 12 59 63 10.1111/j.1523-5378.2007.00565.x 17991178

[B28] Armuzzi A Cremonini F Bartolozzi F Canducci F Candelli M Ojetti V Cammarota G Anti M De Lorenzo A Pola P The effect of oral administration of Lactobacillus GG on antibiotic-associated gastrointestinal side-effects during Helicobacter pylori eradication therapy Aliment Pharmacol Ther 2001 15 163 169 10.1046/j.1365-2036.2001.00923.x 11148433

[B29] Cremonini F Di Caro S Covino M Armuzzi A Gabrielli M Santarelli L Nista EC Cammarota G Gasbarrini G Gasbarrini A Effect of different probiotic preparations on anti-helicobacter pylori therapy-related side effects: a parallel group, triple blind, placebo-controlled study Am J Gastroenterol 2002 97 2744 2749 10.1111/j.1572-0241.2002.07063.x 12425542

[B30] Myllyluoma E Veijola L Ahlroos T Tynkkynen S Kankuri E Vapaatalo H Rautelin H Korpela R Probiotic supplementation improves tolerance to Helicobacter pylori eradication therapy–a placebo-controlled, double-blind randomized pilot study Aliment Pharmacol Ther 2005 21 1263 1272 10.1111/j.1365-2036.2005.02448.x 15882248

[B31] Lionetti E Miniello VL Castellaneta SP Magistá AM de Canio A Maurogiovanni G Ierardi E Cavallo L Francavilla R Lactobacillus reuteri therapy to reduce side-effects during anti-Helicobacter pylori treatment in children: a randomized placebo controlled trial Aliment Pharmacol Ther 2006 24 1461 1468 10.1111/j.1365-2036.2006.03145.x 17032283

[B32] Hurduc V Plesca D Dragomir D Sajin M Vandenplas Y A randomized, open trial evaluating the effect of Saccharomyces boulardii on the eradication rate of Helicobacter pylori infection in children Acta Paediatr 2009 98 127 131 10.1111/j.1651-2227.2008.00977.x 18681892

[B33] Szajewska H Albrecht P Topczewska-Cabanek A Randomized, double-blind, placebo-controlled trial: effect of lactobacillus GG supplementation on Helicobacter pylori eradication rates and side effects during treatment in children J Pediatr Gastroenterol Nutr 2009 48 431 436 10.1097/MPG.0b013e318182e716 19330931

[B34] Goldman CG Barrado DA Balcarce N Rua EC Oshiro M Calcagno ML Janjetic M Fuda J Weill R Salgueiro MJ Effect of a probiotic food as an adjuvant to triple therapy for eradication of Helicobacter pylori infection in children Nutrition 2006 22 984 988 10.1016/j.nut.2006.06.008 16978844

[B35] Tolone C Efficacy of Probinul in reduction of antibiotic gastro-intestinal related side effects in children with respiratory infections Proceeding of 62° National Congress of Italian Society of Pediatrics 2006

[B36] de Bortoli N Leonardi G Ciancia E Merlo A Bellini M Costa F Mumolo MG Ricchiuti A Cristiani F Santi S Helicobacter pylori eradication: a randomized prospective study of triple therapy versus triple therapy plus lactoferrin and probiotics Am J Gastroenterol 2007 102 951 956 10.1111/j.1572-0241.2007.01085.x 17313499

[B37] Fukushima Y Kawata Y Hara H Terada A Mitsuoka T Effect of a probiotic formula on intestinal immunoglobulin A production in healthy children Int J Food Microbiol 1998 42 39 44 10.1016/S0168-1605(98)00056-7 9706796

[B38] Smythies LE Waites KB Lindsey JR Harris PR Ghiara P Smith PD Helicobacter pylori-induced mucosal inflammation is Th1 mediated and exacerbated in IL-4, but not IFN-gamma, gene-deficient mice J Immunol 2000 165 1022 1029 10878379 10.4049/jimmunol.165.2.1022

[B39] del Giudice M De Luca MG The role of probiotics in the clinical management of food allergy and atopic dermatitis J Clin Gastroenterol 2004 38 6 Suppl S84 S85 15220666 10.1097/01.mcg.0000133293.18576.d2

[B40] Del Giudice MM Leonardi S Maiello N Brunese FP Food allergy and probiotics in childhood J Clin Gastroenterol 2010 44 S22 S25 20562632 10.1097/MCG.0b013e3181e102a7

[B41] Hsu PI Wu DC Wu JY Graham DY Is there a benefit extending the duration of Helicobacter Pylori sequential therapy to 14 days? Helicobacter 2011 16 146 152 10.1111/j.1523-5378.2011.00829.x 21435093

